# Retinal Delivery of the Protein Kinase C-β Inhibitor Ruboxistaurin Using Non-Invasive Nanoparticles of Polyamidoamine Dendrimers

**DOI:** 10.3390/pharmaceutics14071444

**Published:** 2022-07-11

**Authors:** Rehab A. Alshammari, Fadilah S. Aleanizy, Amal Aldarwesh, Fulwah Y. Alqahtani, Wael A. Mahdi, Bushra Alquadeib, Qamraa H. Alqahtani, Nazrul Haq, Faiyaz Shakeel, Hosam G. Abdelhady, Ibrahim A. Alsarra

**Affiliations:** 1 Department of Pharmaceutical Sciences, College of Pharmacy, AlMaarefa University, Ad Diriyah 13713, Saudi Arabia; rshammari@mcst.edu.sa; 2 Department of Pharmaceutics, College of Pharmacy, King Saud University, Riyadh 11451, Saudi Arabia; fyalqahtani@ksu.edu.sa (F.Y.A.); wmahdi@ksu.edu.sa (W.A.M.); bquadeib@ksu.edu.sa (B.A.); nazrulhaq59@gmail.com (N.H.); faiyazs@fastmail.fm (F.S.); ialsarra@ksu.edu.sa (I.A.A.); 3 Department of Optometry, College of Applied Medical Sciences, King Saud University, Riyadh 11451, Saudi Arabia; aaldarweesh@ksu.edu.sa; 4 Department of Pharmacology and Toxicology, College of Pharmacy, King Saud University, Riyadh 11451, Saudi Arabia; ghamad@ksu.edu.sa; 5 Department of Physiology & Pharmacology, College of Osteopathic Medicine, Sam Houston State University, 925 City Central Avenue, Conroe, TX 77304, USA; hosam.abdelhady@shsu.edu

**Keywords:** polyamidoamine dendrimers, diabetic retinopathy, protein kinase C-β inhibitor, nanoparticles, ruboxistaurin

## Abstract

Ruboxistaurin (RBX) is an anti-vascular endothelial growth factor (anti-VEGF) agent that is used in the treatment of diabetic retinopathy and is mainly given intravitreally. To provide a safe and effective method for RBX administration, this study was designed to develop RBX nanoparticles using polyamidoamine (PAMAM) dendrimer generation 5 for the treatment of diabetic retinopathy. Drug loading efficiency, and in vitro release of proposed complexes of RBX: PAMAM dendrimers were determined and the complexation ratio that showed the highest possible loading efficiency was selected. The drug loading efficiency (%) of 1:1, 2.5:1, and 5:1 complexes was 89.2%, 96.4%, and 97.6%, respectively. Loading capacities of 1:1, 2.5:1, and 5:1 complexes were 1.6%, 4.0%, and 7.2% respectively. In comparison, the 5:1 complex showed the best results in the aforementioned measurements. The in vitro release studies showed that in 8 h, the RBX release from 1:1, 2.5:1, and 5:1 complexes was 37.5%, 35.9%, and 77.0%, respectively. In particular, 5:1 complex showed the highest drug release. In addition, particle size measurements showed that the diameter of empty PAMAM dendrimers was 214.9 ± 8.5 nm, whereas the diameters of loaded PAMAM dendrimers in 1:1, 2.5:1, 5:1 complexes were found to be 461.0 ± 6.4, 482.4 ± 12.5, and 420.0 ± 7.1 nm, respectively. Polydispersity index (PDI) showed that there were no significant changes in the PDI between the free and loaded PAMAM dendrimers. The zeta potential measurements showed that the free and loaded nanoparticles possessed neutral charges due to the presence of anionic and cationic terminal structures. Furthermore, the safety of this formulation was apparent on the viability of the MIO-M1 cell lines. This nanoformulation will improve the therapeutic outcomes of anti-VEGF therapy and the bioavailability of RBX to prevent vision loss in patients with diabetic retinopathy.

## 1. Introduction

Diabetic retinopathy (DR) is a well-known consequence of poorly controlled hyperglycemia. It is responsible for significant blindness in diabetes mellitus (DM) patients [1]. When DR progresses to a vision-threatening complication, vitreous surgery and laser photocoagulation are the only effective procedures in restoring or maintaining the vision. Nevertheless, the laser-treated retina usually loses its function and develops scarring tissue [2]. The main drug classes used for the management of DR and other DM related complications are anti-vascular endothelial growth factor (anti-VEGF) drugs, protein kinase C (PKC) inhibitors, corticosteroids, and somatostatin analogues [1]. The development of anti-VEGF therapy as a new approach for treating proliferative DR (PDR) has recently been adopted [3]. Anti-VEGF agents have been identified to reduce or prevent neovascularization and vessel drainage through antagonization of VEGF’s effects as they bind to VEGF, and prevent its cellular action [3,4,5,6]. Ranibizumab, bevacizumab, and pegaptanib intravitreal (IVT) injections are currently being used in the treatment of DR [1].

A modern anti-VEGF therapy which relies on using PKC ß-inhibitor has been developed for the treatment of DR. Ruboxistaurin (RBX) is an investigational drug that has recently attracted a great deal of attention as a potent drug for treating DR. It acts as a selective PKC ß-inhibitor that is still pending for the United States Food and Drug Administration (FDA) and European Medicines Agency (EMA) approval. In diabetic rats, RBX has been found to inhibit PKCβ activity when given orally or intravitreally [7,8]. The findings reported by Aldeweesh (2016) demonstrated that RBX is very potent at lowering VEGF release from human retinal macroglial Müller cells (MIO-M1) in the first 24 h under simulated conditions of hyperglycemia. Furthermore, prolonged exposure to RBX resulted in a continued reduction which could indicate the breakdown of VEGF over time [9]. This medicine could slow down or prevent DR progression and which will lead to better quality of life for DM patients. Therefore, this potential candidate needs to be formulated in an appropriate dosage form to be available upon approval by the FDA and EMA [10]. The ideal route for RBX administration can be attained through reaching the retina at a constant rate. In recent years, invasive drug delivery systems (DDSs) have been chosen to deliver anti-VEGFs to the retina, to reduce the risk of systemic side effects and provide better drug targeting for oral and systemic drug delivery [11]. Delivering RBX to the retina via invasive methods can successfully slow down the progression of DR by exposing the retina to a high level of RBX, but unfortunately, will lead to the appearance of unwanted side effects. As a consequence, these side effects will not be tolerated which affects the compliance of patients and thus minimizes the efficacy of the therapy. In this case, microvascular complications of DR remain advanced over time, causing vision to be distorted and quality of life to be reduced. Non-invasive dosage forms, however, are self-administered by the patient and therefore well tolerated as they do not involve injections or invasive equipment. Hopefully, they would increase the patient compliance toward the treatment without experiencing the ocular discomfort and complications associated with IVT injections [12]. If the non-invasive delivery of retinal drugs is achievable and efficient, RBX eye drops could be used as prophylactic approach to lessen the formation of new fragile vessels in the retina [13]. In recent years, researchers have been studying topical drug delivery for their potential in the treatment of retinal diseases [13,14]. The distribution of dexamethasone (DEX) in the ocular tissue was studied by Bessonova et al. (2011). In fact, DEX was found in the eyelid, cornea, aqueous humor, iris, lens, vitreous cavity, retina, and choroid at a range of concentrations following topical application. However, the highest levels of DEX were found in the anterior structures of the eye. This study has shown that DEX cannot be transported to the posterior chamber of the eye via topical application, resulting in a lack of therapeutic responses to treat retinal diseases [14].

Likewise, delivery of oral drugs has been investigated as a non-invasive alternative; however, it has largely been ineffective [15]. Hence, the ideal method is to develop a DDS that can passively deliver RBX from the ocular surface, through the layers of the eye and reside in the retinal tissues [11,12,13]. This can be achieved by the production of nanocarriers that are capable of encasing and delivering therapeutic agents to their site of action [11,12,13]. In the past years, a number of nanocarriers have been investigated for ocular drug delivery, especially degradable nanoparticles (NPs) made with polymers, such as dendrimers, liposomes, polylactic-co-glycolic acid (PLGA) nanoparticles, and chitosan nanoparticles [11,12,13]. These studies suggested that the properties of nanocarriers could influence their application in both segments of the eye [11,12,13]. In fact, dendrimers are exceptional polymers, which offer various advantages over conventional linear or branched polymers [1]. These advantages are better solubility in common solvents, monodisperse distribution, controlled particle size, and higher area/volume ratio [1,12]. These nanocarriers have proved themselves to be very effective to be used as bioavailability, permeability, and solubility enhancers for different routes of administration [1]. For that reason, the goal of this study was to formulate and characterize RBX nanoparticles by encapsulating RBX within the internal cavities of polyamidoamine (PAMAM) dendrimer generation 4.5 and 5, respectively. Out of different generation PAMAM dendrimers, only intermediate generation 3.5–5 dendrimers are suitable for drug delivery carriers. Due to the application of generation 4.5 and 5 as drug delivery carriers, these carriers were developed in this study [12]. Generation 4.5 dendrimers are anionic in nature; however, generation 5 dendrimers are neutral in nature [1,13].

## 2. Materials and Methods

### 2.1. Materials

RBX (LY-333531 hydrochloride, 1 mg vial), dimethyl sulfoxide (DMSO), dialysis cellulose membrane (MWCO 14,000 Da), reusable plastic sample cuvette, folded capillary ζ-cells, and human vascular endothelial growth factor (Hu VEGF) ELISA were obtained from Sigma Aldrich (St. Louis, MO, USA). Polyamidoamine dendrimer generation 4.5 (PAMAM dendrimers G4.5, 1 g vial) and polyamidoamine dendrimer generation 5 (PAMAM dendrimers G5, 1 g vial) were purchased from Nanosynthons (Mt. Pleasant, MI, USA). All chemicals and reagents were of analytical grade.

### 2.2. Formulation of Anionic G4.5 and Neutral G5 Complexes

To achieve loading of RBX into anionic G4.5 and neutral G5 PAMAM dendrimers, RBX (1 mg) was dispersed into 1 mL of dimethyl sulfoxide (DMSO) making a concentration of (0.1% *w*/*v*). The obtained PAMAM dendrimers G4.5 or G5 (1 g) were further dispersed into 10 mL of purified water to achieve a concentration of (10% *w*/*v*). A number of formulations were prepared for the optimization of the best one. In this study, a molar ratio of 1:1, 2.5:1, 5:1, and 25:1 PAMAM dendrimers G4.5: RBX and PAMAM dendrimers G5: RBX were prepared and coded as G4.5 1:1, G4.5 2.5:1, G4.5 5:1, and G4.5 25:1, respectively for G4.5 complexes and G5 1:1, G5 2.5:1, G5 5:1, and G5 25:1, respectively for G5 counterparts. The amounts of these complexes were calculated and added to the reaction mixture. In each one of these mixtures, the volume was completed with deionized water to 1 mL. Briefly, RBX and amounts for each proposed complex are shown in Table 1. After preparation, the reaction mixtures were stirred for 24 h in the dark at 4 °C [16]. The formulated complexes were extracted by removing the excess amounts of the undissolved drug by cold dialysis using cellulose membrane (MWCO 14,000 Da) against distilled water for 24 h [17,18]. The complexes were then lyophilized for 72 h using Labcono Free Zone 6 Liter Benchtop Freeze Dry System and stored at −30 °C for further use [19].

### 2.3. Measurement of Particle Size (PS), Polydispersity Index (PDI), and ζ-Potential

PS and PDI of G4.5 and G5 complexes and the empty PAMAM dendrimers G4.5 and G5 were measured by Zetasizer (Malvern Instruments Ltd., Malvern, UK) [20], which was based on dynamic light scattering (DLS). DLS analysis was performed in triplicate at 25 °C and scattering angle of 90°. The volume-average PS was determined for each sample. The surface charge of G4.5 and G5 complexes and the empty PAMAM dendrimers G4.5 and G5 was determined using Zetasizer (Malvern Instruments Ltd., Malvern, UK) [20]. The ζ-potential of these nanoparticles was determined in an electric field. The velocity and direction of the nanoparticle movement was measured and was demonstrated to be proportional to their ζ-potential. Analysis was performed in triplicate at 25 °C [21].

### 2.4. Determination of Drug Loading Efficiency (DE%)

The loading of different amounts of the drug into the dendritic structure of PAMAM dendrimers G4.5 and G5 was studied to estimate the maximum drug loading efficiency of each complex. To achieve this, DE% of G4.5 and G5 complexes was determined by using the dialysis method. G4.5 and G5 complexes were placed into a cellulose membrane (MWCO 14,000 Da), then immersed in deionized water and stirred for 24 h at 4 °C [22]. After that, a sample from the surrounding media (i.e., deionized water) was withdrawn and measured by Nanodrop UV spectrophotometer at 254 nm. This was carried out to indirectly determine the amount of the drug loaded in PAMAM dendrimers. The measured amount of free drug from the surrounding media was referred as amount of free drug, and the original amount of drug added in the mixture was called amount of total drug. The DE% was calculated by the following equation:

(1) $$\mathrm{DE}\%=\frac{\mathrm{Amount}\;\mathrm{of}\;\mathrm{total}\;\mathrm{drug}-\mathrm{amount}\;\mathrm{of}\;\mathrm{free}\;\mathrm{drug}}{\mathrm{Amount}\;\mathrm{of}\;\mathrm{total}\;\mathrm{drug}}\;\times \;100$$

### 2.5. Transmission Electron Microscopy

The loading visual pattern as well as the morphology of G4.5 and G5 complexes and the empty PAMAM dendrimers, G4.5 and G5, was determined by transmission electron microscopy (TEM) (JEM1230EX; Tokyo, Japan). The image of the nanoparticles was created by using scattered electrons. These electrons were transmitted through the sample, and then the image was formed by detecting the reflected electrons [21].

### 2.6. In Vitro Drug Release of G4.5 and G5 Complexes

In vitro drug release study was carried out to determine the amount of drug released from G4.5 and G5 complexes. To conduct this study, a phosphate buffer solution (PBS) media (pH 7.4) was prepared and used as a dissolution media [23,24]. A total of 200 μL of each studied complex containing 10 μg of RBX was placed into a cellulose membrane with a molecular weight cut-off (MWCO) 14,000 Da, then was immersed in 20 mL of PBS media (pH 7.4). In vitro drug release of G4.5 and G5 complexes was tested at 37 ± 0.5 °C with slow magnetic stirring for a period of 8 h. Aliquots of 500 μL were withdrawn from the solution and replaced with the same volume of fresh PBS at predetermined time points (i.e., 1, 2, 3, 4, 5, 6, 7, and 8 h). After that, RBX amounts in the withdrawn samples was determined by Nanodrop UV spectrophotometer at 254 nm. Throughout the experiments, sink conditions were maintained [1,16].

### 2.7. Stability Studies

According to the manufacturer, PAMAM dendrimers should be stored in the dark at 2–8 °C, while RBX is a light sensitive drug and should be kept at −20 °C. In this work, the effect of light and temperature on the stability of the formulated nanoparticles was investigated. Samples of G4.5 and G5 complexes were stored in the dark and daylight and kept at different temperatures ranging from (4–50) °C as shown in Supplementary Table S1. In addition, the stability of G4.5 and G5 complexes was studied at periods of 1, 3, and 6 months.

#### 2.7.1. Physical Stability

At the end of the 6-month study period, the nanoparticles were observed and signs of color changes, precipitation, or turbidity were recorded [25]. The most physically stable complex was then visualized using scanning electron microscopy (SEM) (JSM 7610F; Tokyo, Japan) to confirm that the complex still preserves its shape and size under certain storage conditions.

#### 2.7.2. Chemical Stability

In addition to the physical stability of G4.5 and G5 complexes, drug content was also assessed under storage conditions mentioned in Table S1. This was carried out by calculating the percent content of RBX remaining in each tested complex. Drug content was quantified by ultra-performance liquid chromatography coupled to the mass spectrometry (UPLC-MS/MS) method as reported in the literature [26].

### 2.8. Cell Culture

#### 2.8.1. Moorfield’s/Institute of Ophthalmology-Müller-1 (MIO-M1) Cells

In this work, cell culture studies were carried out to assess the viability of macroglial Müller cells when exposed to RBX, PAMAM dendrimers, and the proposed complexes as well. The viability of these cells was assessed in controlled and high glucose treatment with and without the tested complexes. The cell line used in this study is MIO-M1; a spontaneously immortalized human cell line. These cell lines were obtained from UCL institute of Ophthalmology, London, UK. These cells were named after the institution where they were isolated, MIO-M1 [27].

#### 2.8.2. Culture of the MIO-M1 Glial Cell Line

MIO-M1 cells were cultured in Dulbecco’s Minimal Essential Medium (DMEM) containing GLUTAMAX and physiological glucose levels of 5.55 mM. The medium was supplemented with 10% fetal bovine serum (FBS) and 50 mg/L penicillin/streptomycin. Prior to experimental work, cells were seeded at 5000 cells per well in 96 well plates (100 μL medium). After that, cells were grown for three days to achieve >80% confluence and were starved with serum-free DMEM for at least 24 h before the experiment [9].

#### 2.8.3. Cell Viability Studies

Cell viability was assessed using (Cell Titer 96 Aqueous Proliferation Assay, Promega, Southampton, UK). This assay was carried out according to the manufacturer’s protocol. This test is a colorimetric assay used to determine the number of viable cells. It is based on the conversion of a 3-(4,5-dimethylthiazol-2-yl)-5-(3-carboxymethoxyphenyl)-2-(4-sulfophenyl)-2H-tetrazolium (MTS) into a brown formazan product when reduced by active cells [28]. The tetrazolium reduction takes place in the mitochondria and measures mitochondrial metabolic rate as a measure of cell viability [29]. Briefly, after exposing cells to experimental conditions, the medium was removed from the 96 well plate and replaced with 100 μL MTS solution. At the end of each tested period (i.e., 24, 48, and 72 h), the fluorescence of formazan was measured at 490 nm with a micro-plate reader [30].

#### 2.8.4. High Glucose Experiments

In this work, MIO-M1 cells were exposed to simulated conditions of hyperglycemia to test the cells’ behavior in the presence of RBX, PAMAM dendrimers, and the proposed complexes. This was achieved by incubating the cells in controlled DMEM (5.55 mM glucose) and high glucose DMEM of (25 mM glucose) for 24, 48, and 72 h with and without the tested compounds. Then, the medium was collected for subsequent cell viability studies [9].

#### 2.8.5. High Glucose Treatment of PAMAM Dendrimers and Complexes

In this study, a drug-dose–response study of RBX, PAMAM dendrimers, and the proposed complexes was carried out in MIO-M1 cells cultured in controlled and high glucose medium. In hyperglycemic conditions, the viability of MIO-M1 cells was assessed using an MTS assay at the end of each tested period (i.e., 24, 48, and 72 h). Two concentrations (200 and 500 nM) of pure RBX were tested. In addition, two concentrations (200 and 500 nM) of RBX formulated as nanoparticles (G4.5 and G5 complexes 25:1) were tested as well. Moreover, PAMAM dendrimers G4.5 and G5 were tested in both cell mediums in the same amount available in G4.5 and G5 complex respectively. This was carried out to examine whether PAMAM dendrimers in the presented concentrations could cause any cytotoxicity to the cells. Furthermore, this step was performed to investigate the safety of the tested compounds in simulated conditions of hyperglycemia and also, to determine the most tolerable doses by MIO-M1 cells.

#### 2.8.6. In Vitro Permeability Study

The integrity of the monolayer cell membrane of MIO-M1 was evaluated via an in vitro permeability study on Transwell^®^ and transendothelial electrical resistance (TEER). The in vitro studies were performed in a 24-well plate format on a polyester membrane (PET) Transwell^®^ (0.33 cm^2^, 0.4 μm pore size). MIO-M1 cells were seeded at a density of 0.5 × 10^6^ cells/insert in the apical (upper) compartment with 300 μL media, and the basolateral (lower) compartment was filled with 900 μL media. The cells were grown in Dulbecco’s Modified Eagle’s medium (DMEM) containing glutamine supplemented with 10% fetal bovine serum and 1% penicillin-streptomycin. MIO-M1 cells were incubated at 37 °C at 5% CO_2_, along with DMEM media of well and insert replenished every two days [31,32].

It is important that MIO-M1 make confluent monolayers so that they require monolayer integrity and will function as a barrier. To evaluate monolayer integrity, the measurement of TEER was performed using an endothelial volt-ohmmeter (EVOM) (World Precision Instruments, Sarasota, FL, USA) according to the manufacturer’s instructions. The resistance value (R _Blank_) in units of (Ω) measured for each insert (no cells) was subtracted from the resistance (R _Total_) across the cell layer on the membrane to obtain the cell specific resistance (R _MIO-M1_):

R _MIO-M1_ (Ω) _=_ R _Total_ − R _Blank_ (2)

TEER resistance (Ω·cm^2^) = R _MIO-M1_ (Ω) × SA _semipermeable membrane surface Area_ (cm^2^) (3)

R _MIO-M1_ value was then multiplied by the surface area of the insert (0.33 cm^2^) to obtain the value of TEER resistance of MIO-M1 (Ω·cm^2^). Each filter was measured three times, and each culture condition was carried out at least with triplicate filters [33,34,35,36].

At the time of the experiments, MIO-M1 was cultured for at least 14 days to reach the highest and constant TEER value of the membrane. Transwells^®^ were washed twice with sterile PBS without calcium and magnesium and transferred to a serum-free medium for the duration of the experiments. At 37 °C in 5% CO_2_ in a humidified incubator, pure drug (RBX), or dendrimers (with or without RBX), at 500 nM of RBX concentration, was incubated for 24 h. The monolayer membrane’s integrity was evaluated by quantifying tracer permeability of solution containing fluorescein isothiocyanate conjugate (FITC)-dextran at an initial concentration of 100 μg/mL, which was added to the apical (upper) compartment. At 1, 3, 6, 12, and 24 h, samples were drawn from the basolateral compartment, which was replaced with an equal volume of fresh medium each time. The fluorescence intensities were measured using a Synergy H1 plate reader (BioTek) at emission/excitation wavelengths of 495/520 nm and were converted to the concentrations of FITC-dextran with the calibration curve [31,32,37,38,39]. The apparent permeability coefficient (Papp) was calculated based on the following equation [38,40]:

Papp (cm/s) = J/(AC_D_) (4)

where, J is the rate of appearance of the compounds in the receiver compartment, A is the surface area of the filter membrane, and C_D_ is the initial concentration in the donor compartment.

### 2.9. Statistical Analysis

Values were expressed as the means and standard error of the mean (SEM). Differences among treated groups and control (MIO-M1 only) were evaluated by one-way analysis of variance (ANOVA) followed by *post-hoc* comparisons using Tukey’s multiple comparisons using Graph Pad Prism (version 9.0, GraphPad Inc., La Jolla, San Diego, CA, USA). A *p* value of 0.05 or less was considered statistically significant.

## 3. Results

### 3.1. Characterization of G4.5 Complexes

#### 3.1.1. Measurement of PS and PDI

PS and PDI measurements were carried out for both G4.5 complexes and the empty PAMAM dendrimers G4.5 and results of PS and PDI for the G4.5 complexes and the empty PAMAM dendrimers G4.5 are demonstrated in Table 2, Figure S1A and Figure S2A, respectively. According to the manufacturer, PS of free PAMAM G4.5 is 143.0 ± 19.6 nm. Results obtained from Zetasizer demonstrated that PS of the empty PAMAM dendrimers G4.5 was 186.0 ± 2.3 nm. When loaded with RBX, the measurements revealed that PS of G4.5 complexes 1:1, 2.5:1, 5:1, and 25:1 was increased to 367.0, 416.0, 289.0, and 301.0 nm, respectively. However, G4.5 complex 25:1 possessed less PS among all studied complexes. PAMAM dendrimers possess lower PDI when compared to other nanoparticles. PDI was measured for G4.5 complexes and the empty PAMAM dendrimers G4.5 and revealed that there is no significant change in PDI measurement of the dendrimers after loading it with RBX (*p* > 0.05). However, G4.5 complex 25:1 possessed the least PDI among all studied nanoformulations (PDI ≤ 0.35).

#### 3.1.2. Measurement of ζ-Potential of G4.5 Complexes

ζ-potential measurements were carried out for G4.5 complexes and the empty PAMAM dendrimers G4.5, and results are demonstrated in Table 2. In this study, ζ-potential measurements demonstrated that ζ-potential of empty PAMAM dendrimers G4.5 is −44.0 ± 2.0 mV. After the loading process, ζ-potential measurements of G4.5 complexes 1:1, 2.5:1, 5:1 and 25:1 was −16.2, −5.1, −6.4, and −13.0 mV, respectively. In fact, the surface charge of PAMAM dendrimers plays an important role in permeability of these nanoparticles through the negatively charged ocular barriers. In this work, it was found that the loaded G4.5 complex 25:1 have preserved its negative charge after the loading process. In summary, the previous features demonstrated with G4.5 complex 25:1 are needed for the non-invasive ocular delivery of RBX.

#### 3.1.3. Determination of DE%

The DE% of G4.5 complexes were determined and results are presented in Figure S3A. In this study, DE% of the studied nanoparticles was similar. However, G4.5 complex 25:1 showed the best DE% among all the other proposed complexes though statistically not significant.

#### 3.1.4. In Vitro Drug Release of G4.5 Complexes

**Figure 1:**
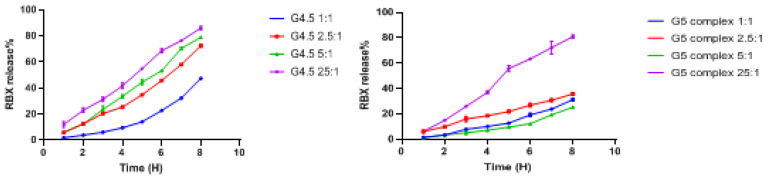
In vitro release profiles of different complexes (mean ± SD, *n* = 6).

Release profiles of G4.5 complexes were studied in order to evaluate the amount of RBX that will be released following the topical instillation of the nanoparticles. As indicated in Figure 1, most of the G4.5 complexes presented a sustained release of RBX. It was shown that at pH 7.4 in a period of 8 h, RBX release of G4.5 complexes 1:1, 2.5:1, and 5:1 was 47.3%, 72.5%, and 79.0%, respectively. In particular, it was observed that the RBX release rate of G4.5 complexes 25:1 was distinctly higher than all the other studied complexes. For a period of 8 h, this complex possessed an initial release of 11.9%. After that, RBX release was gradually increased in a sustained manner up to 86.1%. Moreover, the previous in vitro characterizations of the proposed complexes demonstrated that G4.5 complex 25:1 has revealed the best in vitro release as well as the highest DE% among all other proposed nanoparticles. Because of these remarkable results of G4.5 complex 25:1, this complex was chosen for the next experiments.

#### 3.1.5. Stability Studies of G4.5 Complex

##### Physical Stability

**Figure 2:**
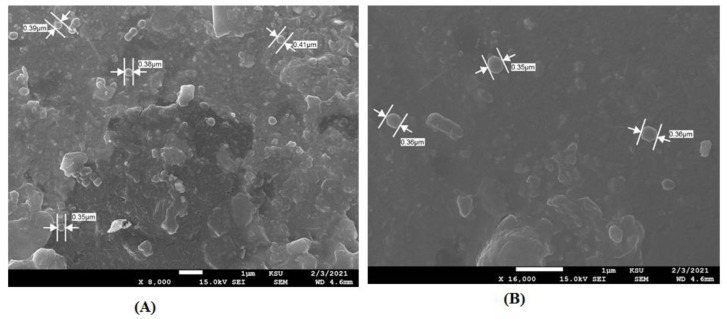
Scanning electron microscope (SEM) overview image of (**A**) G4.5 complex 25:1 and (**B**) G5 complex 25:1.

The stability of the G4.5 25:1 was studied at daylight and in the dark in different temperature conditions ranging from (4–50 °C). For a period of 6 months, the nanoformulations were stored in colorless Eppendorfs and observed for any sign of color change, precipitation, or turbidity. Results are presented in Table S2. After the study period ended, the nanoformulations stored in the dark at 4 °C were examined by the eye inspection and did not show any signs of physical change. In daylight, the sample kept at 25 °C was visualized and showed a color change from pink to colorless while the sample kept in the dark did not show any physical changes. Moreover, a precipitate and a color change were also observed at higher temperatures, 37 °C and 50 °C, respectively. The previous investigation indicated that it is suitable to store the nanoparticles in amber containers at 4 °C. In addition, these nanoparticles were visualized using SEM to assess their shape and size. As seen in Figure 2A, the complex possessed an almost spherical shape and particle size equivalent to those measured by Zetasizer Nano ZS.

##### Chemical Stability

The present work is a quantitative study of the stability of RBX to assess the effect of light source and temperature overtime. This test will help in choosing the best storing conditions for the nanoparticles. Samples of G4.5 complex 25:1 containing 1 μg of RBX was stored at the dark and daylight at different temperatures (4–50 °C). Each one of these samples was kept for 1, 3, and 6 months and analyzed by UPLC MS/MS after the end of each tested period. To determine percent content of RBX for each studied sample, the remaining amount of RBX was quantified from the equation of calibration curve using peak area values and then percent content of RBX was calculated. Results obtained from the stability studies are shown in Figure S4A. Data indicated that RBX is more stable when stored in amber containers. Upon protecting the nanoformulation from light, higher percent content of RBX was demonstrated. In addition, it was observed that the stability of RBX in PAMAM dendrimers G4.5 was slightly decreased with time. In a duration of 6 months, measurements of percent content of RBX in G4.5 complex 25:1 remain above 80.0% in this time period. Moreover, data show that RBX is more stable within PAMAM dendrimers at 4 > 25 > 37 > 50 °C. In fact, the highest percent content of RBX was achieved when storing the nanoparticles in the dark at 4 °C.

##### TEM

**Figure 3:**
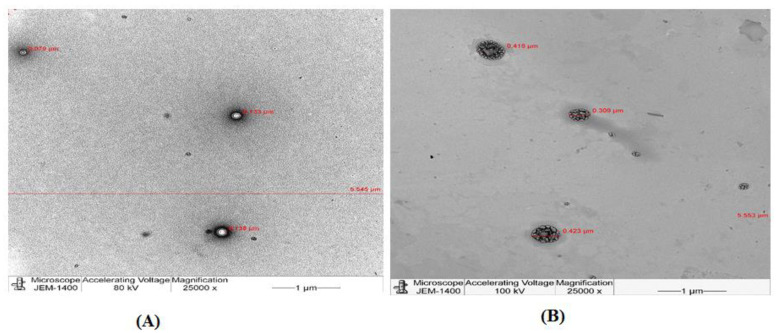
Transmission electron microscope (TEM) overview images of (**A**) empty PAMAM dendrimers G4.5 and (**B**) G4.5 complex 25:1.

G4.5 complex 25:1 and the empty PAMAM dendrimers G4.5 were visualized by TEM to determine their morphology and to confirm the loading of RBX. Figure 3 displays the morphology of empty PAMAM dendrimers G4.5 as well as G4.5 complex 25:1.

### 3.2. Characterization of G5 Complexes

#### 3.2.1. Measurement of PS and PDI

PS and PDI measurements were carried out for both G5 complexes and the empty PAMAM dendrimers, and results are demonstrated in Table 2, Figure S1B, and Figure S2B. According to the manufacturer, the PS of free PAMAM G5 dendrimers is 224.0 ± 13.0 nm. Results obtained from Zetasizer demonstrated that PS of the empty PAMAM dendrimers G5 was 214.9 ± 8.5 nm. When loaded with RBX, the measurements revealed that PS of G5 complexes 1:1, 2.5:1, 5:1, and 25:1 was increased to 461.2, 482.4, 669.8 and 307.1 nm, respectively. Indeed, PS increase of loaded PAMAM dendrimers may be attributed to RBX entrapment within the internal cavities of PAMAM dendrimers. However, G4.5 complex 25:1 possessed the lowest PS among all studied nanoformulations. PDI was measured for the free and loaded PAMAM dendrimers and revealed that there are no significant changes in PDI of the dendrimers after loading it with RBX except for G5 complex 5:1. In fact, G5 complex 25:1 possessed the least PDI among all studied nanoformulations (PDI ≤ 0.39). However, a significant increase in PDI was demonstrated with G5 complex 5:1 (Table 2 and Figure S2B).

#### 3.2.2. Measurements of ζ-Potential of G5 Complexes

ζ-potential provided by the manufacturer indicated that the neutral PAMAM G5 dendrimers possesses a neutral charge. In this study, ζ-potential measurements demonstrated that the surface charge of the empty PAMAM dendrimers G5 was −0.1 ± 0.0 mV. Table 2 demonstrates ζ-potential of G5 complexes. After the loading process, ζ-potential of G5 complexes 1:1, 2.5:1, 5:1, and 25:1 was 4.3, 5.5, 9.7, and −0.0 mV, respectively. Indeed, the free and loaded PAMAM G5 dendrimers possessed a neutral charge even after the loading process. Results indicated that ζ-potential of PAMAM dendrimers G5 were not affected by the drug loading process (*p* > 0.05).

#### 3.2.3. Determination of DE%

DE% of the studied nanoparticles showed similar percent content between G5 complexes. Although not statistically significant, G5 complex 25:1 showed the highest DE% among all the other proposed complexes. Results of DE% are presented in Figure S3B.

#### 3.2.4. In Vitro Drug Release of G5 Complexes

As indicated in Figure 1, nearly all G5 complexes possessed a sustained release of RBX. It was shown that at pH 7.4 in a period of 8 h, in vitro release of G5 complexes 1:1, 2.5:1, and 5:1 was 31.3%, 35.8%, and 25.3%, respectively. In particular, RBX release rate of G5 complexes 25:1 was distinctly higher than all the other studied complexes. For a period of 8 h, this complex possessed an initial release of 6.3%. After that, RBX release was gradually increased in a sustained manner up to 81.0%. In fact, among the proposed complexes, G5 25:1 has demonstrated the best in vitro characterizations. Because of these remarkable results of G4.5 complex 25:1, the next studies were manipulated for this complex.

#### 3.2.5. Stability Studies of G5 Complex

##### Physical Stability

The stability of the G5 25:1 was studied at daylight and in the dark in different temperature conditions ranging from (4–50 °C). For a period of 6 months, the nanoformulations were stored in colorless Eppendorfs and observed for any sign of color change, precipitation, or turbidity. Results are presented in Table S3. This study was performed to investigate which storing condition is suitable for G5 complex 25:1. Based on the result of this investigation, it is clearly indicated that G5 complex 25:1 must be stored in amber containers at 4 °C. In addition, these nanoparticles were visualized using SEM to assess their shape and size. As seen in Figure 2B, the complex possessed an almost spherical shape and particle size equivalent to those measured by Zetasizer Nano ZS (Malvern, UK).

##### Drug Content

The results for the stability of RBX in G5 25:1 complex were similar to the results obtained from the G4.5 complex 25:1 stability study. It was noticed that there were no significant differences in RBX peak area between G4.5 and G5 samples. Percent content of RBX in G5 complex 25:1 was calculated and demonstrated in Figure S4B.

##### TEM

**Figure 4:**
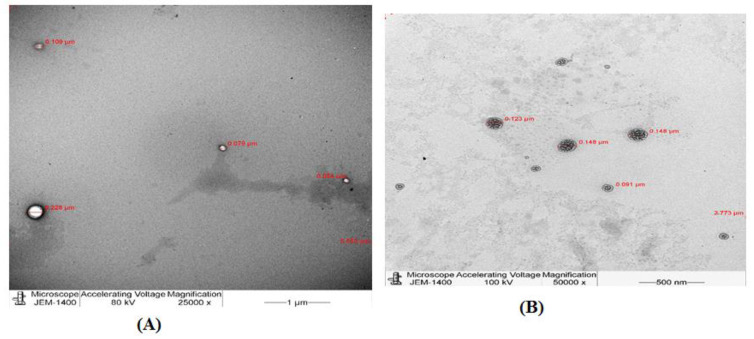
TEM overview images of (**A**) empty PAMAM dendrimers G5 and (**B**) G5 complex 25:1.

G5 complex 25:1 and the empty PAMAM dendrimers G5 were visualized by TEM to determine their morphology and to confirm the loading of RBX. Figure 4 displays the morphology of empty PAMAM dendrimers G5 as well as G5 complex 25:1 complex.

### 3.3. Cell Viability Studies (MTS Assay)

#### 3.3.1. Dose–Response Studies in Controlled Mediums

##### Dose–Response of RBX

**Figure 5:**
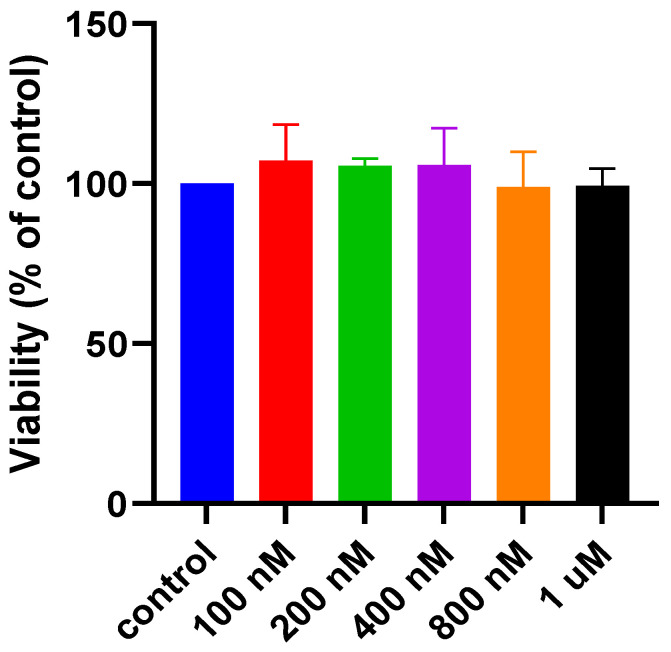
Effect of RBX after 24 h exposure on the cell viability of MIO-M1 cells under controlled conditions (mean ± SD, *n* = 3).

In this work, the safety of RBX was assessed by cell viability studies using MIO-M1 cell line. These investigations were carried out using MTS assay. MIO-M1 cells were cultured in a controlled glucose medium (5.55 mM glucose media) and treated with a range of doses of RBX (100 nM–1 μM). After 24 h, viability (%) of the treated cells was assessed and compared to the viability of untreated cells that were cultured in the same controlled conditions. As seen in Figure 5, viability of the treated cells with RBX did not show any significant reduction when compared to viability of untreated cells (*p* > 0.05). This finding indicates that RBX is safe and the investigated doses are tolerable by MIO-M1 cell line in normal conditions.

##### Dose–Response of PAMAM Dendrimers G4.5

**Figure 6:**
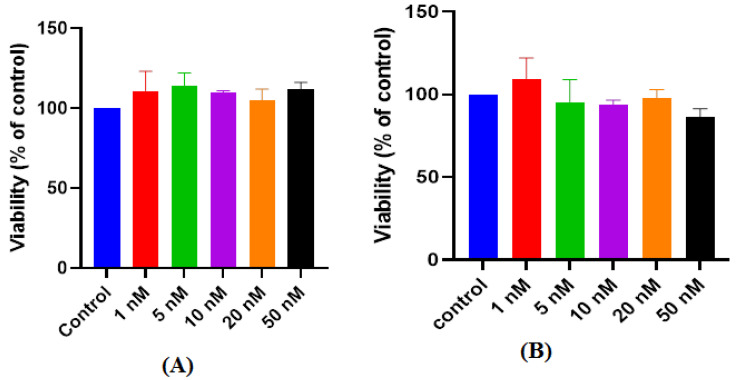
Effect of (**A**) PAMAM dendrimers G4.5 and (**B**) PAMAM dendrimers G5 after 24 h. Exposure on the cell viability of MIO-M1 cells under controlled conditions (mean ± SD, *n* = 3).

Similar to the previous work, the safety of PAMAM dendrimers G4.5 was assessed using different concentrations ranging from (1–50 nM). These investigations were carried out using MTS assay. After 24 h, viability of the treated cells was assessed and compared to the viability of untreated cells that were cultured in the same controlled conditions. As seen in Figure 6A, viability of the treated cells with PAMAM dendrimers G4.5 did not show any significant reduction when compared to viability of untreated cells (*p* > 0.05). These finding indicates that PAMAM dendrimers G4.5 is safe and the investigated doses are tolerable by MIO-M1 cell line in normal conditions.

##### Dose–Response of PAMAM dendrimers G5

Similar to the previous work, the safety of PAMAM dendrimers G5 was assessed using different concentrations ranging from (1–50 nM). These investigations were carried out using MTS assay. After 24 h, viability of the treated cells was assessed and compared to the viability% of untreated cells that were cultured in the same controlled conditions. As seen in Figure 6B, viability of the treated cells with PAMAM dendrimers G5 did not show any significant reduction when compared to viability of untreated cells (*p* > 0.05). These findings indicate that the PAMAM dendrimer G5 is safe and the investigated doses are tolerable by MIO-M1 cell line in normal conditions.

##### Dose–Response of G4.5 Complex 25:1

**Figure 7:**
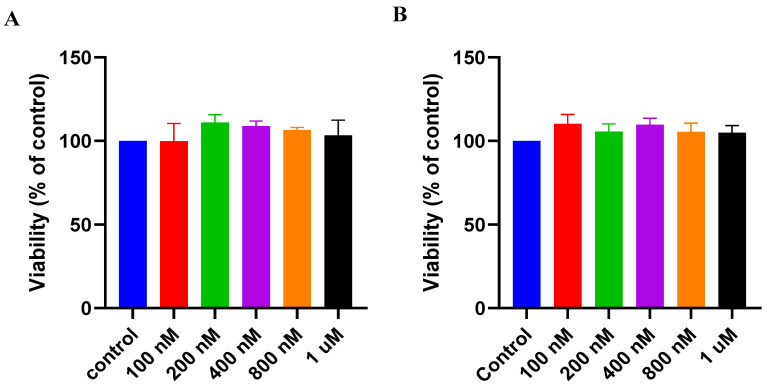
Effect of G4.5 complex 25:1 (**A**) and G5 complex 25:1 (**B**) after 24 h exposure on the cell viability of MIO-M1 cells under controlled conditions (mean ± SD; *n* = 3).

It was clearly indicated that RBX and PAMAM dendrimers are well tolerated by MIO-M1 cells. More investigations were carried out to assess the safety of the formulated G4.5 complex 25:1. This was carried out using MTS assay. After 24 h, viability of the treated cells was assessed using different concentrations ranging from (100 nM–1 µM) and compared to the viability of untreated cells that were cultured in the same controlled conditions. As seen in Figure 7A, viability of the treated cells with PAMAM dendrimers G4.5 did not show any significant reduction when compared to the viability of untreated cells (*p* > 0.05). These finding indicates that G4.5 complex 25:1 is safe and the investigated doses are tolerable by MIO-M1 cell line in normal conditions.

##### Dose–Response of RBX G5 Complex 25:1

Similarly, the safety of G5 complex 25:1 was assessed using MTS assay. After 24 h, viability of the treated cells was assessed using different concentrations ranging from (100 nM–1 µM) and compared to the viability of untreated cells that were cultured in the same controlled conditions. As seen in Figure 7B, viability of the treated cells with G5 complex 25:1 did not show any significant reduction when compared to viability of untreated cells (*p* > 0.05). This finding indicates that G5 complex 25:1 is safe and the investigated doses are tolerable by MIO-M1 cell line in normal conditions.

#### 3.3.2. Effect of High Glucose Treatment on the Viability of MIO-M1 Cells

**Figure 8:**
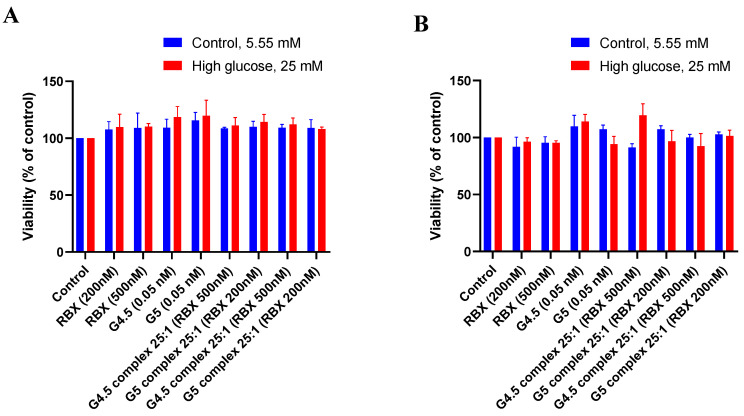
Effect of the tested compounds after (**A**) 24 h and (**B**) 48 h. Exposure on the cell viability of MIO-M1 cells under controlled and high glucose mediums (mean ± SD, *n* = 3).

A series of experiments were conducted to test the effect of RBX, PAMAM dendrimers, and the proposed complexes on the viability of MIO-M1 cells in two different culture mediums; the controlled medium (5.55 mM) and high glucose medium were approximately five times normal levels of glucose to simulate hyperglycemia for MIO-M1 cells (25 mM). To test the effect of the tested compounds in high glucose treatment, MIO-M1 cells were incubated with the tested compounds for 24, 48, and 72 h. Then, viability was calculated and compared to the relevant controlled medium of that particular time point. As seen in Figure 8, in the presence of the tested compounds, there is no significant loss in cell viability of MIO-M1 cells cultured in high glucose media when compared to relevant controlled media of each studied time point (*p* > 0.05).

#### 3.3.3. In Vitro Permeability Study

**Figure 9:**
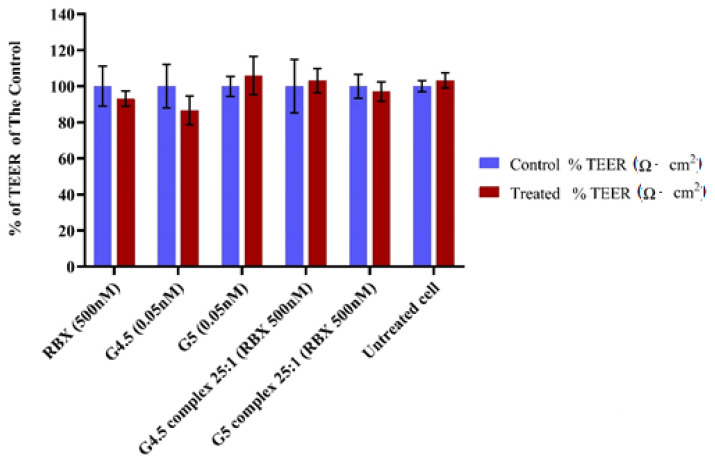
TEER measurements of monolayer cell membrane 24 h after exposure to treatments. Data represent % of TEER mean of the control ± SEM (*n* = 3–4).

**Figure 10:**
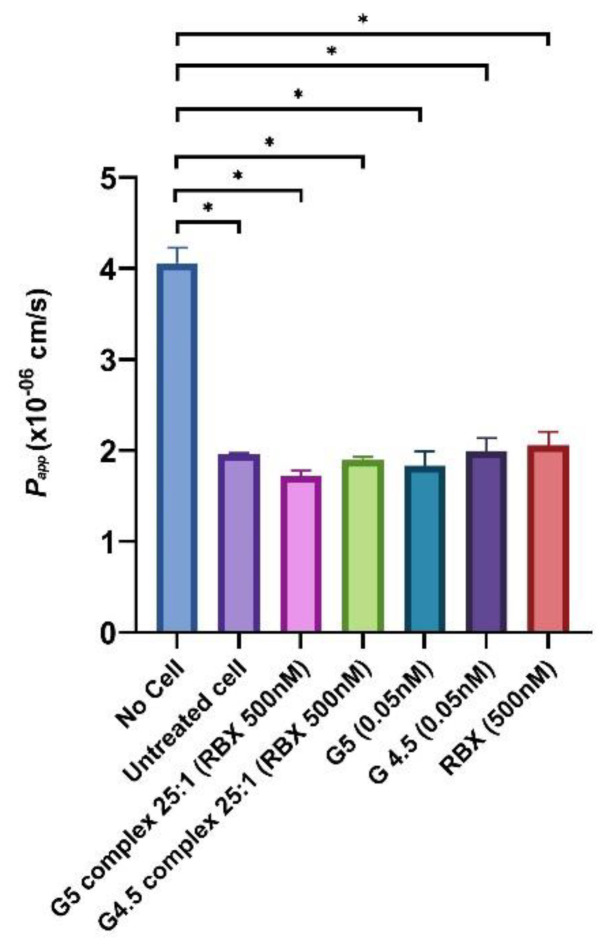
Apparent permeability coefficient (Papp) of the MIO-M1 monolayer cell after 24 treatments * *p* value < 0.01.

The effect of RBX and dendrimers (with or without RBX) on the monolayer integrity of MIO-M1 was examined by measuring TEER values across the cell monolayer membrane in the upper Transwell^®^ chamber before and after treatments (Figure 9). At the beginning of this experiment, the MIO-M1 monolayer was developed on a porous membrane of Transwell^®^ inserts before TEER values were measured to evaluate the monolayer integrity. TEER values across the cell monolayer were measured every other day until the cell monolayers exhibited constant TEER values of more than 8 Ω*cm^2^. These values were used as controls for each particular insert. A total of 500 nM of RBX and dendrimers (with or without RBX) was incubated for 24 h, and then TEER values were measured again to investigate the influence of complexes on monolayer integrity of MIO-M1 cells as compared to RBX and dendrimers alone. A paired *t*-test was used to determine whether there was a statistically significant difference between TEER values when the MIO-M1 monolayer was treated compared to non-treated cells. As highlighted in Figure 9, TEER values were shown to be approximately 13.0 %, which is the highest reduction (9.7 ± 1.7→8.4 ± 0.7) across the cell monolayer membrane following 24 h of treatment with G4.5 (0.05 nM). Even though the TEER values of G4.5 (0.05 nM) showed the highest reduction across the cell monolayer membrane, it was not statistically significant when compared to TEER values before treatment at a *p*-value less than 0.01. Indeed, the reduction in TEER value of MIO-M1 monolayer, following the treatment of G5 complex 25:1, G4.5 complex 25:1, and RBX, was not statistically significant according to paired student *t*-test (*p* value < 0.01). As detailed in Figure 10, the data exhibited a high Papp (4.0 ± 0.1 × 10^−6^ cm/s) of the groups without the MIO-M1 monolayer. However, the Papp (1.9 ± 0.0 × 10^−6^ cm/s) of the developed MIO-M1 without treatment was hardly distinguishable from that observed by Papp of G5 complex and G4.5 complex, at the same condition, were 1.8 ± 0.0 × 10^−6^ cm/s and 1.7 ± 0.0 × 10^−6^ cm/s, respectively. In the in vitro permeability assessment, the collected data of cell treated with G5 complex and G4.5 complex showed that the permeability of MIO-M1 did not significantly change compared to that of non-treated MIO-M1 cells. One-way ANOVA analysis revealed that no statistically significant differences were detected among the permeability of the MIO-M1 monolayer with or without treatment of G5 complex, G4.5 complex, and RBX alone. Further statistical analysis by a *post-hoc* test showed that the difference between dendrimers with or without RBX on MIO-M1 permeability was not statistically significant at a *p* value < 0.01.

## 4. Discussion

PAMAM dendrimers have recently been studied to determine their ability to provide an effective and noninvasive drug delivery to the retina. Recent findings of Chang Liu et al. (2016) found that PAMAM dendrimers could rapidly penetrate from the surface of the eye into the vitreous and resided in the retina. This new approach is considered to be important in the development of a safe and noninvasive drug delivery for posterior segment diseases [41]. Herein, we aimed to develop a retinal delivery system by which RBX would be passively delivered from the surface of the eye to the retina. This was achieved through the development of RBX nanoparticles using PAMAM dendrimers. To achieve our aim, several complexes were formulated using 2 different generations and surface functional groups (i.e., anionic PAMAM dendrimers G4.5 and neutral PAMAM dendrimers G5). Then, these complexes were characterized and assessed for their cytotoxicity effects using the MIO-M1 cell line. The ideal nanosystem for ocular therapy formulations must have high loading efficiency, small particle size, and fast drug release [42]. In the current study, the diameter of PAMAM G4.5 and G5 was found to be 186.6 ± 2.3 and 214.9 ± 8.5 nm, respectively, supported by the findings of Yavuz (2015) [1]. Furthermore, nanoparticles with a size range typically < 400.0 nm are suitable for ophthalmic use according to Gorantla et al. (2020) [43]. In addition, the results of this study revealed that the mean particle size values of G4.5 and G5 loaded with RBX varied from 289.4 ± 39.9 nm to 482.4 ± 28.7 nm, indicating the loaded polymer is suitable as an ophthalmic formulation. Results indicated that the proposed complexes have significantly increased particle size when compared to the empty PAMAM dendrimers (*p* < 0.05), which is attributed to entrapment of RBX molecules within the internal cavities of PAMAM dendrimers. Sizes of both complex formulations of each generation were enlarged in a range approximately between 100.0–280.0 nm with the exception of PAMAM G5 5:1 complex which was measured as 669.8 ± 35.3 nm. High particle size of this formulation might be a result of aggregation. Such results were in line with the findings of Yavuz et al. [1].

The PDI is usually measured to determine the heterogeneity of a sample based on the size. PDI is the potential result of size distribution or aggregation of the particles in a sample during isolation or analysis [44,45]. It was demonstrated that PDI values of empty PAMAM dendrimers G4.5 and G5 were 0.297 ± 0.055 and 0.350 ± 0.008, respectively. Surfing the literature revealed that PDI < 0.35 is suitable for ocular drug delivery [46]. G4.5 and G5 complexes possessed a PDI value ranging from (0.335–0.394). Such findings revealed that there are no significant changes in PDI of the dendrimers after loading it with RBX (*p* > 0.05). Findings of this work were indicative of monodispersity of PAMAM dendrimers. Specifically, G4.5 and G5 complex 25:1 possessed the least PDI among all studied nanoformulations (PDI ≤ 0.35) indicating high particle homogeneity. In fact, the lower PDI value is much closer to achieving a monodisperse system; so, the latter complexes were considered the most monodisperse systems that is suitable for ocular drug delivery of RBX. However, an exception to the aforesaid findings was observed with G5 complex 5:1. This complex possessed a significantly high PDI value (0.587 ± 0.106), which was previously discussed in possessing a high particle size as well. This observation could be related to the aggregation of the nanoparticles. According to Tawfik et al. (2018), PDI measurements of all studied vardenafil/PAMAM complexes were characterized by small PDI values ≤ 0.25 [21]. Another study by Peng et al. demonstrated that the PDI of the PAMAM formulation was found to be in a range of (0.230–0.339), which supports our findings [47].

ζ-potential measurements determine the electrostatic potential at the electrical double layer surrounding a nanoparticle in a solution. In fact, ζ-potential plays an important role in determining the stability of nanoparticles as well as knowing the intensity of electrostatic attraction between biomolecules and the nanoparticles. Nanoparticles with a ζ-potential between −10.0 and +10.0 mV are considered approximately neutral, while nanoparticles with ζ-potentials of greater than +30.0 mV or less than −30.0 mV are considered to be strongly cationic and strongly anionic, respectively. Since most of eye cellular membranes are negatively charged, ζ-potential can affect a nanoparticle’s tendency to permeate membranes, with cationic particles generally displaying more toxicity associated with cell wall disruption [48]. A study carried out by Yavuz (2015) found that ζ-potential values of blank dendrimer generations G4.5 was strongly anionic (−45.9 + 8.8) [1]. On the other hand, nanosynthons; manufacturer of PAMAM dendrimers G5, has stated that PAMAM dendrimers G5 possesses a neutral charge as they were synthesized with aminoethanol surface. In the current work, the ζ-potential measurement of G4.5 was −44.0 mV, which is similar to the result demonstrated by Yavuz [1]. Additionally, it was found that the surface charge of G5 was −0.2 mV which is in line with the information provided by nanosynthons. In this work, measurements of ζ-potential for G4.5 and G5 complexes showed that, ζ-potential values, either negative or neutral, were increased in the presence of RBX. Moreover, G4.5 complexes 25:1 and 1:1 remained anionic in contrast to PAMAM dendrimers used in complex 2.5:1 and 5:1, which exhibited a neutral charge after the loading process. Results obtained from this work also showed that zeta potential values for neutral complexes (i.e., G5 complexes) increased yet did not significantly change after the loading process (*p* > 0.05). However, it was clear that the loading of RBX, a weakly basic drug, into PAMAM dendrimers had a positive impact on these ζ-potential findings. Results demonstrated by Tawfik et al. (2018) supported our findings as vardenafil, a weakly basic drug (similar to RBX), was encapsulated within PAMAM dendrimers and ζ-potential measurements were increased after the loading process [21]. Drugs, such as RBX, could be encapsulated or conjugated to dendrimers. The morphology of G4.5 and G5 complexes was observed by TEM. It was shown that complexes as well as blank PAMAM dendrimers showed a discrete spherical morphology. Investigation of drug–polymer binding revealed that RBX was encapsulated within PAMAM dendrimers and no binding to PAMAM surface occurred. Furthermore, the ability of a fixed amount of RBX to be incorporated into various dendrimer generations and concentrations was investigated to estimate DE%. The drug loading efficiency is influenced by certain factors. These include the nature of the polymer, the encapsulated drug molecules, and polymer–drug ratio [25]. DE% of RBX in G4.5 and G5 complexes varied from 88.8% to 98.68%. It was demonstrated that drug loading efficiency of RBX did not show any significant differences among the proposed nanoparticles (*p* > 0.05). However, increasing the amount of RBX did not significantly show any differences compared to lower concentrations in the proposed nanoparticles (*p* > 0.05). Nabavizadeh et al. (2016) prepared different concentrations of 5:1, 5:2, 5:3, 5:4, and 5:5 of capecitabine: PAMAM dendrimers and studied DE% of these formulations. They found that DE% of capecitabine decreased with higher PAMAM dendrimer concentration. Increasing PAMAM dendrimer concentration has led to stronger electrostatic interactions between capecitabine and PAMAM dendrimers as a result [22,23]. In contrast to the findings reported by the later study, no significant differences occurred between DE% of G4.5 complexes as well as G5 complexes. Our findings were not similar to Nabavizadeh et al. (2016) results and were not affected by the factor of conjugation [22]. We supported our findings by visualizing the loading pattern of the complexes under TEM, which showed that RBX was only encapsulated within PAMAM dendrimers and not conjugated to PAMAM surface. Tawfik et al. (2018) studied vardenafil DE% among the proposed nanoparticles and revealed that direct correlation between the dendrimer concentration and vardenafil DE% was not held true for all concentrations. Statistically, no significant differences in vardenafil DE% were observed between the proposed nanoformulations [21]. These finding are in line with our results, which showed no significant differences in DE% were observed between 1:1, 2.5:1, 5:1, and 25:1 RBX: PAMAM.

Based on the results of DE%, the appropriate ratio of RBX-PAMAM dendrimer was selected for stability studies and ex vivo cell line studies, which was found to be 25:1 for each studied PAMAM generation. Moreover, Yavuz et al. (2015) studied the in vitro drug release of dexamethasone nanoparticles using different generations of PAMAM dendrimers. Since the nanoparticles are expected to be cleared from the eye in approximately an hour, it was desired that dexamethasone should be immediately released to penetrate to the posterior segment of the eye. Thus, a 3 h long in vitro drug release study was conducted in PBS at 37 °C. In a period of 3 h, the studied complexes released dexamethasone in a range of 40.0 to 80.0% [1]. In our work, a period of 8 h in vitro release study was carried out since the complex formulations were designed as fast release nanoparticles to be applied topically and their ocular retention time is suggested to be shorter. After the release study period has ended, it was found that G4.5 complexes released RBX in a range from 47.0–86.0%, while G5 complexes released RBX in a range of 25.0–81.0%. Cumulative release (%) of RBX from the studied nanoparticles showed that the drug was released in a sustained manner. In this study, it was clearly noticed that the highest release of RBX was demonstrated with G4.5 complex 25:1 and G5 complex 25:1 up to 86.0% and 81.0% respectively *(p* < 0.05). The release of RBX from the suggested complexes was compared to other in vitro release studies of PAMAM dendrimers. Gothwal et al. (2017) formulated bendamustine nanoparticles of PAMAM dendrimers G4. In this study, PAMAM dendrimers released the drug in a sustained manner up to 72 h [49,50]. Another study presented by Yesil-Celiktas et al. (2017) reported that PAMAM dendrimers provided a sustained release of BCA for six consecutive days [51]. All of these studies are in line with our findings of this study which demonstrated that PAMAM dendrimers are found to provide a sustained release of RBX in all studied formulations. In another study, Kesharwani et al. (2015) observed that the curcumin release decreased as PAMAM generation increased despite having a higher loading capacity [52]. The result of this study supports our findings in which RBX was released in higher percentages from the lower generation (G4.5) when compared to RBX release from the higher generation (G5).

Furthermore, the stability of G4.5 and G5 25:1 complexes was inspected visually to evaluate the appearance of any color change, precipitation, or turbidity. This was carried out to determine the disintegration of the proposed nanoparticles when exposed to light and/or high temperatures. Results obtained from this work showed that after the study period has ended, it was clearly observed that with increasing the storage temperature, physical changes such as color change and precipitation were recorded. Our findings revealed that the nanoformulations were found to be physically stable at 4 °C, whether kept in the dark or the light. Shadrack et al. (2015) has investigated physical stability of tetramethylscutellarein nanoparticles using PAMAM dendrimers at different temperature conditions (i.e., 0, 27 and 40 °C) for two months. Investigations showed a sign of color change, turbidity and precipitate formation were observed for formulations stored in light indicating that light had an influence on their stability [25]. These findings come in agreement with results obtained from our study that suggested that light exposure have can influence the stability of RBX nanoparticles. Findings of the later study found that nanoparticles were relatively stable when kept in the dark at high temperature (i.e., 40 °C). In contrast to these findings, we have observed physical changes of the nanoformulation at higher temperatures even when kept in the dark. This phenomenon was described by Prajapati et al. (2009) who explained that the formation of precipitates observed may be attributed to the opening of dendritic structures at higher temperatures [53]. By that means, we suggest that RBX complexes should be kept in the dark at 4 °C to provide the maximum stability of the proposed formulations. Also, another measurement was carried out to investigate the stability of RBX in the proposed complexes when exposed to different storage conditions. When each study period has ended, drug content was determined in each studied complex by using UPLC MS/MS. After that, percent content of RBX was calculated to estimate the loss of RBX in each tested complex. Results obtained from UPLC-MS/MS showed that RBX in G4.5 complex 25:1 remained above 90% stability at 4 °C for 6 months, the stability was slightly decreased over time from one month to 6 months. Moreover, percent content of RBX was apparently decreased by more than 20% with increasing the temperature. The results of this experiment revealed that the percent content of RBX is higher at 4 > 25 > 37 > 50 °C. Similar finding were observed with G5 complex 25:1. All studied nanoformulations were more stable when stored in the dark despite the influence of temperature over time. As a result of these observations, it is suggested that RBX nanoparticles should be stored in amber containers at 4 °C for maximum stability. RBX is a very sensitive drug that should be kept away from light and higher temperatures. To avoid RBX degradation, this drug must be stored in an amber container at −20 °C. In this work, it was observed that formulating RBX in a nanoparticulate system using PAMAM dendrimers can improve the stability of RBX in addition to its tendency to provide a facile retinal drug delivery.

Furthermore, ex vivo cell line studies were conducted to evaluate the safety of the formulated nanoparticles in MIO-M1 cells. It was found that the drug RBX in a range of concentrations did not affect the cell viability in both controlled and high glucose mediums. Similar results were observed with the work of Aldarwesh (2016) [9]. Also, empty and loaded PAMAM dendrimers G4.5 and G5 were investigated for their cytotoxicity effect in MIO-M1 cell line. The nanoparticles, in different concentrations, did not affect the viability of these cells, despite the type of cell medium (i.e., controlled or high glucose medium). To our knowledge, this is the first cytotoxicity study that was carried out to assess ex vivo behavior of PAMAM dendrimers using the MIO-M1 cell line. To assess the negative effect of complexes on the MIO-M1 monolayer integrity as a simple blood–retinal barrier (BRB) model, an in vitro permeability study on Transwell^®^ and TEER was carried out. The cells were developed on the upper surface of the semi-permeable membrane in the Transwell^®^ culture chambers in order to quantify the disruption level of new complexes on a monolayer of MIO-M1 cells. The difference in the TEER values of dendrimers and complexes was not statistically significant compared to TEER values before treatment at a *p*-value less than 0.01. Indeed, the reduction in TEER value of MIO-M1 monolayer, following the treatment of G5 complex 25:1, G4.5 complex 25:1, RBX, was not statistically significant according to paired Student *t*-test (*p* < 0.01). These findings suggest that both G5 complex 25:1 and G4.5 complex 25:1 did not significantly damage the monolayer integrity of MIO-M1 cells. The value of apparent permeability of developed MIO-M1 monolayers was confirmed by comparing it to the literature value for MIO-M1 cells [54]. Furthermore, the permeability values of about 10–6 cm/s have been previously observed in various in vitro BRB studies [32,38]). After confirmation of the monolayer integrity of MIO-M1, the monolayer integrity of MIO-M1 was inspected by calculating Papp of the barriers using FITC-dextran. During in vitro permeability assay, the monolayer cells were exposed to one of the G5 complex 25:1, G4.5 complex 25:1, and RBX for 24 h. The effect of the complexes on the barrier integrity was examined and compared to RBX, non-treated cells, and no cell groups with regards to the impact of treatment on the apparent permeability coefficient of the MIO-M1 monolayer. As shown in Figure 10, the Papp value of non-seeded insert was significantly higher than the Papp value of untreated cell, indicating the formation of MIO-M1 monolayer membrane on the Transwell^®^. Furthermore, the results showed no statistically significant differences among the permeability value of the MIO-M1 monolayer with or without treatment of dendrimers and complexes. The difference between dendrimers with or without RBX on MIO-M1 permeability was not statistically significant (*p* ˃ 0.05). These findings suggest that both G5 complex and G4.5 complex did not have a negative effect on the monolayer integrity of MIO-M1 cells. In addition, the above mentioned data support the cell viability studies which indicate the safety of G5 complex and G4.5 complex on MIO-M1 cell line in normal conditions.

## 5. Conclusions

RBX is a newly potent anti-VEGF drug that has been recently investigated for the treatment of DR. At present, anti-VEGF therapy is usually delivered to the retina via intravitreal injections carrying many ocular complications that cause patient noncompliance. To provide the maximum therapeutic outcomes of treating diabetic retinopathy, a non-invasive therapy of RBX was designed. This was carried out using nanoparticles of PAMAM dendrimers. Several methodologies have been implemented and the non-invasive nanoparticles that show the best in vitro characterization were formulated and characterized. The proposed nanoparticles will overcome ocular complications associated with the invasive therapy of diabetic retinopathy and the result will help in increasing patient adherence. The future direction of this study is to assess the in vivo behavior of RBX nanoparticles and to evaluate their permeability properties and ability to provide non-invasive retinal delivery of the potent anti-VEGF, RBX.

## Figures and Tables

**Table 1:** Calculated amounts of G4.5 and G5 complexes.

Formulation	Amount of RBX (0.1% *w*/*v*)	Amount of PAMAM Dendrimers G4.5 (10% *w*/*v*)	Amount of PAMAM Dendrimers G5 (10% *w*/*v*)
G4.5 complexes			
G4.5 complex 1:1	50 μL	25 μL	-
G4.5 complex 2.5:1	50 μL	10 μL	-
G4.5 complex 5:1	50 μL	5 μL	-
G4.5 complex 25:1	50 μL	1 μL	-
G5 complexes			
G5 complex 1:1	50 μL	-	28 μL
G5 complex 2.5:1	50 μL	-	11.2 μL
G5 complex 5:1	50 μL	-	5.6 μL
G5 complex 25:1	50 μL	-	1.1 μL

**Table 2:** PS, PDI and ζ-potential of blank and loaded PAMAM dendrimers G4.5 and G5 (mean ± SD, *n* = 3).

RBX-PAMAM Nanoparticles	PS (nm) ± SD	PDI ± SD	ζ-Potential in (mV) ± SD
Empty PAMAM dendrimers G4.5	186.0 ± 2.3	0.297 ± 0.040	−44.0 ± 2.0
G4.5 complex 1:1	367.0 ± 13.0	0.335 ± 0.010	−16.2 ± 3.1
G4.5 complex 2.5:1	416.0 ± 4.3	0.337 ± 0.030	−5.1 ± 10.4
G4.5 complex 5:1	289.0 ± 14.9	0.361 ± 0.030	−6.4 ± 1.8
G4.5 complex 25:1	301.0 ± 7.1	0.304 ± 0.010	−13.0 ± 1.6
Empty PAMAM dendrimers G5	214.0 ± 8.5	0.356 ± 0.010	−0.2 ± 0.0
G5 complex 1:1	461.0 ± 6.4	0.394 ± 0.010	4.3 ± 1.3
G5 complex 2.5:1	482.0 ± 9.5	0.388 ± 0.020	5.5 ± 0.2
G5 complex 5:1	669.0 ± 2.0	0.587 ± 0.100	9.7 ± 4.9 **
G5 complex 25:1	307.0 ± 6.9	0.380 ± 0.030	−0.0 ± 0.0

** *p* ≤ 0.01.

## Data Availability

Not applicable.

## Supplementary Materials

The following supporting information can be downloaded at: https://www.mdpi.com/article/10.3390/pharmaceutics14071444/s1, Figure S1: Particle size (PS) of (A) G4.5 and (B) G5 complexes along with the empty PAMAM dendrimers G4.5 and G5 (mean ± SD, *n* = 3; ANOVA* *p* ≤ 0.05); Figure S2: Polydispersity index (PDI) of (A) G4.5 complexes and (B) G5 complexes along with the empty PAMAM dendrimers G4.5 and G5 (mean ± SD, *n* = 3; ANOVA* *p* ≤ 0.05); Figure S3: Drug loading efficiency (DE%) of (A) G4.5 complexes and (B) G5 complexes (mean ± SD, *n* = 3; ANOVA* *p* ≤ 0.05); Figure S4: Stability studies of RBX in (A) G4.5 complexes and (B) G5 complexes under different storage conditions (mean ± SD, *n* = 3); Table S1: Different storage conditions used to assess the stability of G4.5 and G5 complexes; Table S2: Effect of heat and light on the stability of G4.5 complex 25:1; Table S3: Effect of heat and light on the stability of G5 complex 25:1.
