# Supplementary material for: Retinal Delivery of the Protein Kinase C-β Inhibitor Ruboxistaurin Using Non-Invasive Nanoparticles of Polyamidoamine Dendrimers
Source: Pharmaceutics. 2022 Jul 11;14(7):1444. doi: 10.3390/pharmaceutics14071444 (PMC9322846; doi:10.3390/pharmaceutics14071444)
Supplement: Supplementary file 1 [file pharmaceutics-14-01444-s001.zip › pharmaceutics-1778569-supplementary.pdf]

Supplementary materials

Table S1. Different storage conditions used to assess the stability of G4.5 and G5 complexes.

Nanoformulation	Condition	Temperature (°C)
G4.5 or G5 complex	At daylight	4
	In the dark	
	At daylight	25
	In the dark	
	At daylight	37
	In the dark	
	At daylight	50
	In the dark	

Table S2. Effect of heat and light on the stability of G4.5 complex 25:1.

Nanoparticles	Condition	Temperature (°C)	Color change	Precipitation	Turbidity
G4.5 complex 25:1	At daylight	4	-	-	-
	In the dark		-	-	-
	At daylight	25	√	-	-
	In the dark		-	-	-
	At daylight	37	-	√	-
	In the dark		-	√	-
	At daylight	50	√	-	-
	In the dark		√	-	-

Table S3. Effect of heat and light on the stability of G5 complex 25:1.

Nanoparticles	Condition	Temperature (°C)	Color Change	Precipitation	Turbidity
G5 complex 25:1	At daylight	4	-	-	-
	In the dark		-	-	-
	At daylight	25	√	-	-
	In the dark		-	-	-
	At daylight	37	-	√	-
	In the dark		-	√	-
	At daylight	50	√	√	-
	In the dark		√	-	-

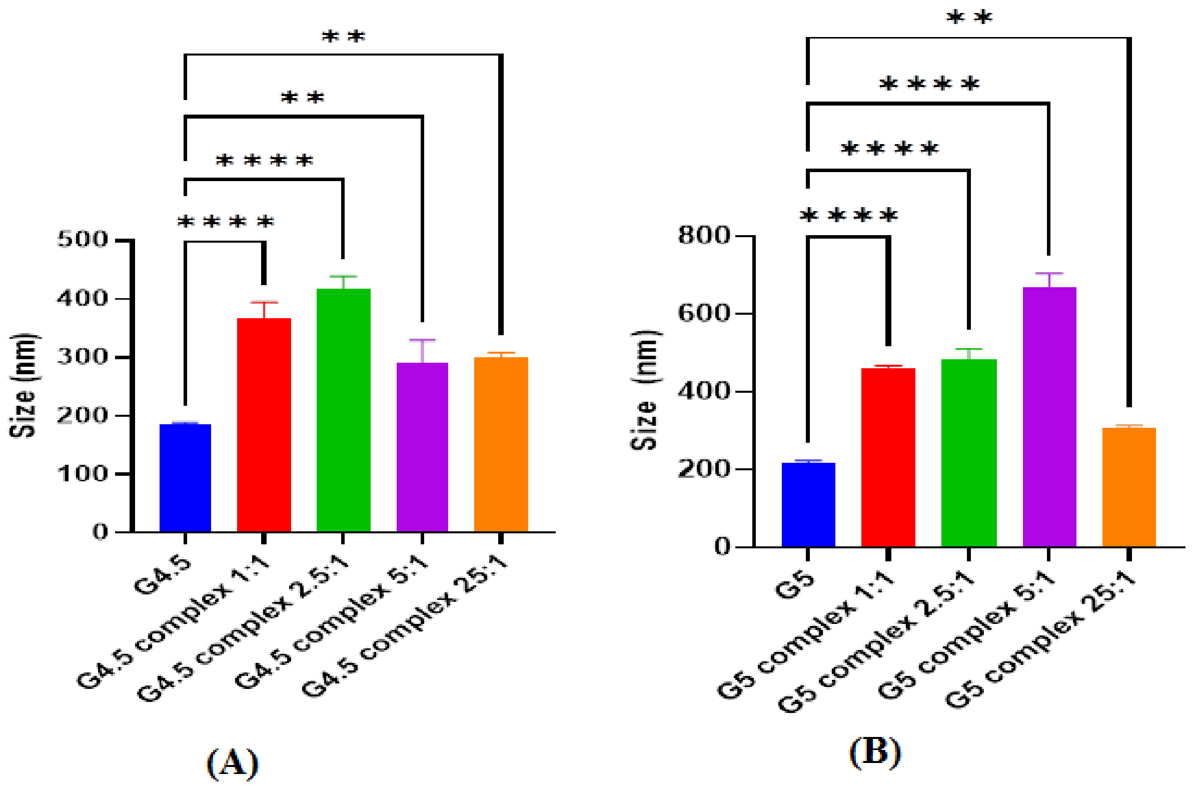

Figure S1. Particle size (PS) of (**A**) G4.5 and (**B**) G5 complexes along with the empty PAMAM dendrimers G4.5 and G5 (mean ± SD, $n = 3$; ANOVA, ** $p \leq 0.01$, **** $p \leq 0.0001$).

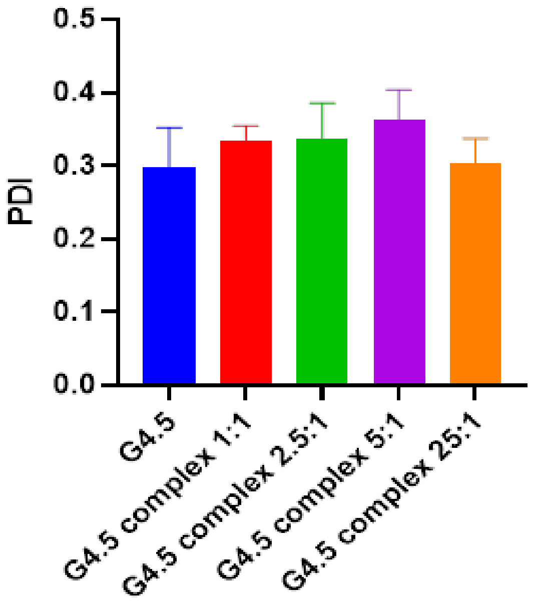

(A)

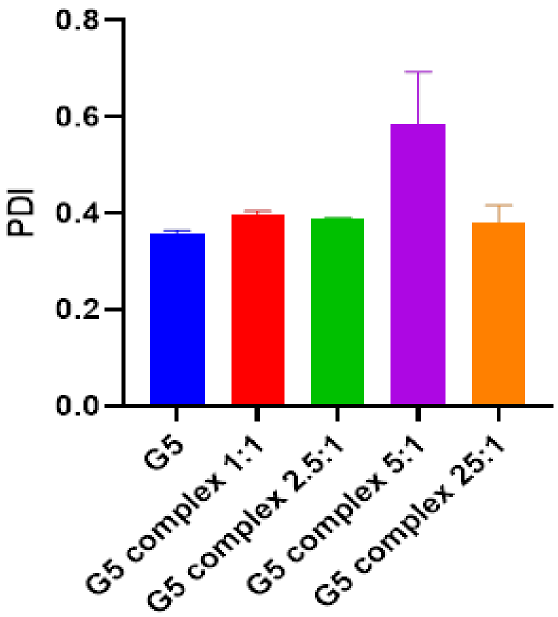

(B)

Figure S2. Polydispersity index (PDI) of (**A**) G4.5 complexes and (**B**) G5 complexes along with the empty PAMAM dendrimers G4.5 and G5 (mean ± SD, n = 3).

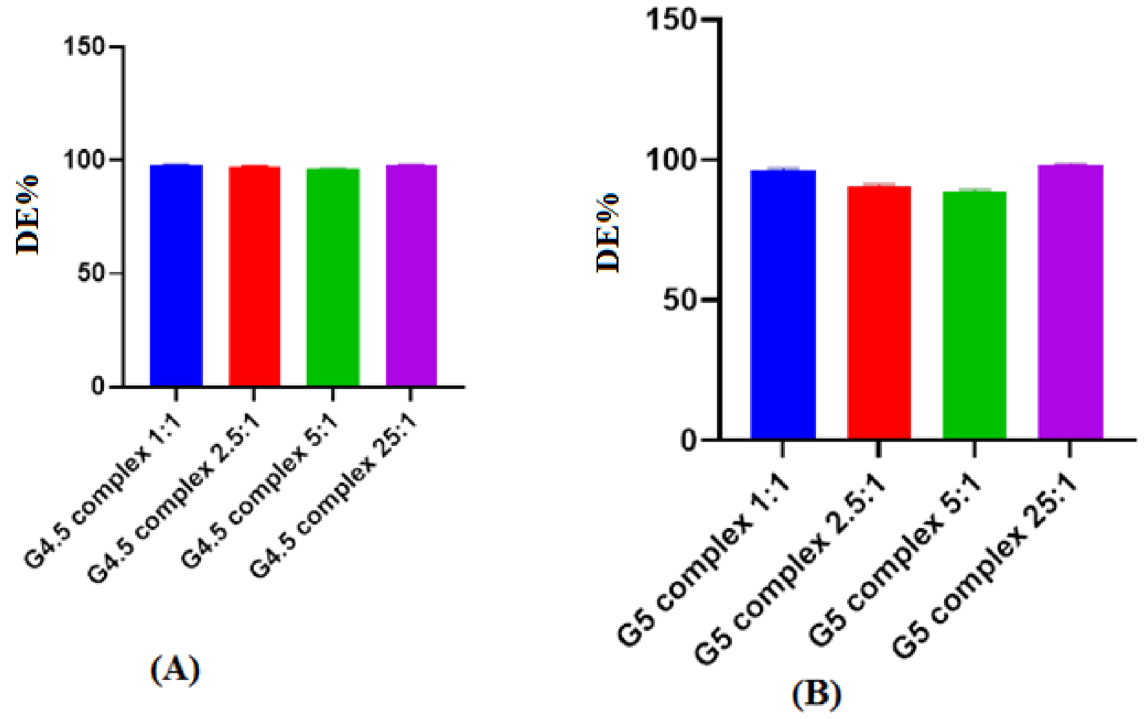

Figure S3. Drug loading efficiency (DE%) of (**A**) G4.5 complexes and (**B**) G5 complexes (mean ± SD, *n* = 3).

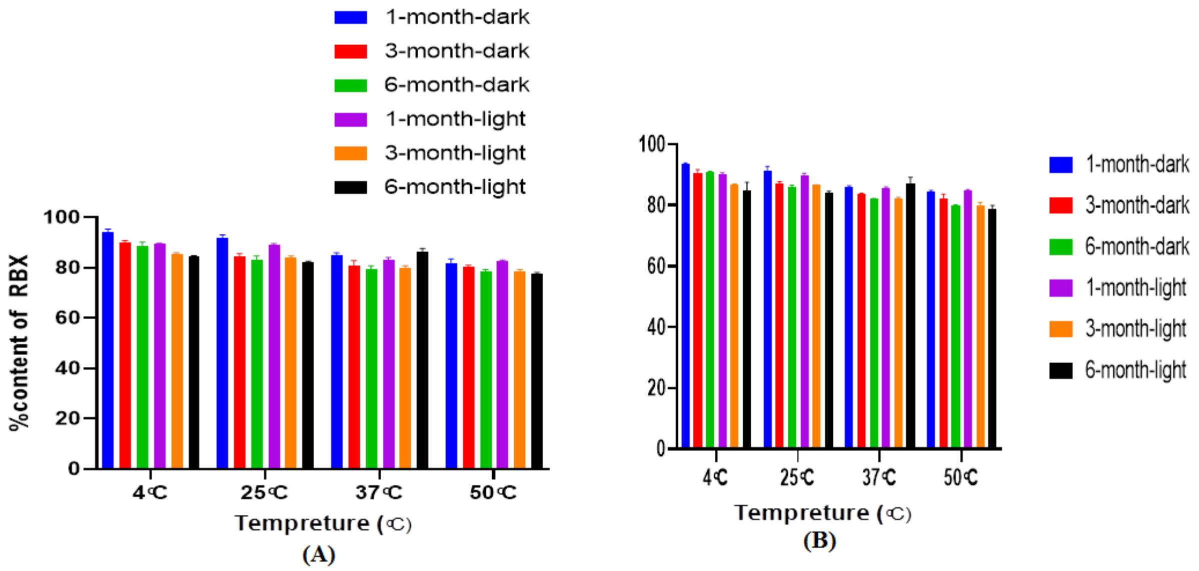

Figure S4. Stability studies of RBX in (**A**) G4.5 complexes and (**B**) G5 complexes under different storage conditions (mean ± SD, n = 3).
